# An exploration of the influence of diagonal dissociation and moderate changes in speed on locomotor parameters in trotting horses

**DOI:** 10.7717/peerj.2190

**Published:** 2016-06-30

**Authors:** Sarah Jane Hobbs, John E.A. Bertram, Hilary M. Clayton

**Affiliations:** 1 Centre for Applied Sport and Exercise Sciences, University of Central Lancashire, Preston, Lancashire, United Kingdom; 2 Department of Cell Biology and Anatomy, Cumming School of Medicine, University of Calgary, Calgary, Alberta, Canada; 3 Sport Horse Science, LC, MI, United States

**Keywords:** Stability, Balance, Turning moments, Temporal, Timing, Trunk, Pitch, Trot, Locomotion

## Abstract

**Background.** Although the trot is described as a diagonal gait, contacts of the diagonal pairs of hooves are not usually perfectly synchronized. Although subtle, the timing dissociation between contacts of each diagonal pair could have consequences on gait dynamics and provide insight into the functional strategies employed. This study explores the mechanical effects of different diagonal dissociation patterns when speed was matched between individuals and how these effects link to moderate, natural changes in trotting speed. We anticipate that hind-first diagonal dissociation at contact increases with speed, diagonal dissociation at contact can reduce collision-based energy losses and predominant dissociation patterns will be evident within individuals.

**Methods.** The study was performed in two parts: in the first 17 horses performed speed-matched trotting trials and in the second, five horses each performed 10 trotting trials that represented a range of individually preferred speeds. Standard motion capture provided kinematic data that were synchronized with ground reaction force (GRF) data from a series of force plates. The data were analyzed further to determine temporal, speed, GRF, postural, mass distribution, moment, and collision dynamics parameters.

**Results.** Fore-first, synchronous, and hind-first dissociations were found in horses trotting at (3.3 m/s ± 10%). In these speed-matched trials, mean centre of pressure (COP) cranio-caudal location differed significantly between the three dissociation categories. The COP moved systematically and significantly (*P* = .001) from being more caudally located in hind-first dissociation (mean location = 0.41 ± 0.04) through synchronous (0.36 ± 0.02) to a more cranial location in fore-first dissociation (0.32 ± 0.02). Dissociation patterns were found to influence function, posture, and balance parameters. Over a moderate speed range, peak vertical forelimb GRF had a strong relationship with dissociation time (*R* = .594; *P* < .01) and speed (*R* = .789; *P* < .01), but peak vertical hindlimb GRF did not have a significant relationship with dissociation time (*R* = .085; *P* > 0.05) or speed (*R* = .223; *P* = .023).

**Discussion.** The results indicate that at moderate speeds individual horses use dissociation patterns that allow them to maintain trunk pitch stability through management of the cranio-caudal location of the COP. During the hoof-ground collisions, reduced mechanical energy losses were found in hind-first dissociations compared to fully synchronous contacts. As speed increased, only forelimb vertical peak force increased so dissociations tended towards hind-first, which shifted the net COP caudally and balanced trunk pitching moments.

## Introduction

The trot is regarded as a symmetrical gait with the limbs moving by diagonal pairs (Alexander, 1984; Hildebrand, 1965; Lee, Bertram & Todhunter, 1999) but slow motion analysis in horses has indicated that the diagonal footfalls often occur with some degree of contact asynchrony (Clayton, 1994; Deuel & Park, 1990; Drevemo et al., 1980; Holmström, Fredricson & Drevemo, 1994; Weishaupt et al., 2010). The asynchrony of this footfall sequence is reported to vary between horses (Deuel & Park, 1990; Holmström, Fredricson & Drevemo, 1995) and, depending on the hoof contact sequence, this can be classified as synchronous, hind-first or fore-first dissociation. In dressage horses there is a positive association between hind-first contacts and subjective assessment of gait quality at trot (Holmström, Fredricson & Drevemo, 1994). Differences in the pattern and timing of dissociations have also been noted in horses performing advanced dressage movements. One such movement is passage, which is described as a very collected, elevated, cadenced and graceful trot (Fédération Equestre Internationale, 2016) with a particularly high step and body carriage. When performing passage, longer relative hind-first dissociation times were found by Weishaupt et al. (2010) compared to collected trot. Untrained differences in dissociation have also been found in dogs. A tendency toward hind-first dissociation has been reported in trotting Greyhounds while Labrador Retrievers tend toward fore-first dissociation (Bertram et al., 2000). The breed-specific dissociation patterns may arise to balance differences in body motions resulting from differences in conformation (body/limb proportion and mass distribution). These subtle differences in contact timing also influence the timing of peak force production in the diagonal pairs of limbs (Weishaupt et al., 2010). As such, dissociation may have important consequences on trotting dynamics, particularly as moments around the centre of mass (COM) are most affected by the vertical force components and their effective distance to the COM (Hobbs, Richards & Clayton, 2014).

Diagonal synchronization can provide trunk pitch and roll stability if the load distribution between the limbs remains consistent (Hildebrand, 1985). In horses at the trot, the head and trunk are rotationally stabilized and this helps to determine and maintain whole-body spatial orientation (Dunbar et al., 2008). In this context, trunk stability is defined as minimization of roll and pitching moments about the COM. During trotting, activation of *longissimus dorsi* and *rectus abdominis* muscles increases spinal stiffness (Robert et al., 2002), which provides a stable platform for limb articulation and force transmission (Nauwelaerts & Clayton, 2009; Robert et al., 2002). Hind-first contacts were thought to reflect nose-up pitch rotation of the trunk, with the forequarters elevated relative to the hindquarters (Holmström, Fredricson & Drevemo, 1994). Whether trunk inclination or stability is affected by asynchronous foot contacts is currently unknown.

The mechanical effects of diagonal dissociation have not been explored in detail and may be important to locomotor efficiency. Mechanical energy losses through collision-like deflection of the animal’s mass have been identified as a major source of mechanical cost during locomotion (Ruina, Bertram & Srinivasan, 2005). Limb contact timing and sequence have a substantial effect on the magnitude of collisional losses. For instance, during galloping collisional losses are reduced by using a limb contact sequence that distributes changes in the COM angular deflection between limbs, thereby decreasing the net deflection angle during each contact (Bertram & Gutmann, 2009; Ruina, Bertram & Srinivasan, 2005). The effect of the limbs has been likened to a rolling rimless wheel, in which a larger number of spokes acting in sequence allows the system to roll more effectively (Ruina, Bertram & Srinivasan, 2005). Although the footfall contact and lift off sequencing during trotting is far more discrete compared to galloping, it may still be advantageous to dissociate the diagonal footfalls to reduce mechanical losses.

The aims of this study were to: (1) to investigate the mechanical effects of different dissociation patterns in a larger group of horses trotting at the same speed; (2) using a smaller group of horses, to assess which of these mechanical effects are most influenced by changes in speed; and (3) evaluate potential reasons why individual horses adopt a predominant diagonal dissociation pattern. We anticipate that: (1) hind-first dissociation increases with speed to overcome the tendency to accumulate forward and upward residual moments around the COM due to increasing forelimb forces; (2) diagonal dissociation reduces collisional energy losses; and (3) within horse dissociation predominance is evident at the horse’s preferred trotting speed. Alterations in footfall timing reduce collisional losses in ring-tailed lemurs (  O’Neill & Schmitt, 2012) and are used to adjust the centre of pressure (COP) location in running cockroaches as speed increases (Ting, Blickhan & Full, 1994). Speed-dependent effects on collisional losses, stability and balance do not appear to have been reported in trotting. Furthermore, it is not known whether footfall patterns are associated with specific mechanical effects that are independent of trotting speed.

## Methods

The study was performed with approval from the institutional animal care and use committee, Michigan State University, USA under protocol number 02/08-020-00. All horses were ridden regularly and had received basic dressage training but none was trained to a medium or advanced level. Horses were judged by a veterinarian who was experienced in lameness evaluation to be sound at trot with lameness grade <1 on a 0–5 scale (American Association of Equine Practitioners, 1991). Horses were accustomed to the laboratory environment before data collection commenced and were trained to trot in hand at steady state velocity along the runway and over the force platforms.

## Experimental Data Collection

Kinematic data were recorded using 10 infra-red cameras (Eagle cameras, Motion Analysis Corp.) and motion analysis software (Cortex 1.1.4.368; Motion Analysis Corp., Santa Rosa, CA, USA). Force data were recorded with a threshold of 50 N using four synchronized force plates arranged linearly with their long axes parallel to the runway. The first and last plates measured 60 × 120 cm (FP61290; Bertec Corporation, Columbus, OH, USA) and the two middle plates measured 60 × 90 cm (FP6090, Bertec Corporation). In 14 horses, kinematic data were collected at 120 Hz and force data at 960 Hz. In the other 4 horses, kinematic data were collected at 100 Hz and force data at 1,000 Hz to facilitate synchronization with accelerometers worn by this subset of horses. The camera system and force platforms were positioned mid way along a 40 m runway, which allowed steady state gait to be obtained prior to data capture. All horses were led by a handler, who ran with the horses through the data collection volume with a loose rope to ensure they did not interfere with the animal’s natural gait. The horses were trained to match their speed with that of the handler.

Reflective cubic markers were attached to the horse’s skin (Hobbs, Richards & Clayton, 2014), but with one additional mid-segment tracking marker on the left and right antebrachial and crural segments to improve the estimation of their position during the trotting trials (Cappello et al., 1997).

### Speed matched data

20–30 trotting trials were collected from seventeen horses of mixed breed with (mean ± s.d.) height 1.50 ± 0.06 m and mass 465 ± 34 kg. Successful trials were those in which the horse moved straight and consistently through the data collection volume with a diagonal pair of hooves making valid contacts with different force plates. Dissociations were classified as hind-first, synchronous or fore-first for each diagonal separately. One successful, speed-matched (3.3 m/s ± 10%) trial per horse was selected, which included one left LFRH and one right RFLH diagonal.

### Speed range data

A further ten successful trotting trials per horse were collected from five horses of mixed breed with (mean ± s.d.) height 1.50 ± 0.03 m and mass 455 ± 19 kg. These trials were performed at a speed that each horse favoured, which represented a narrow range of speeds for each horse.

### Procedure

**Figure 1:**
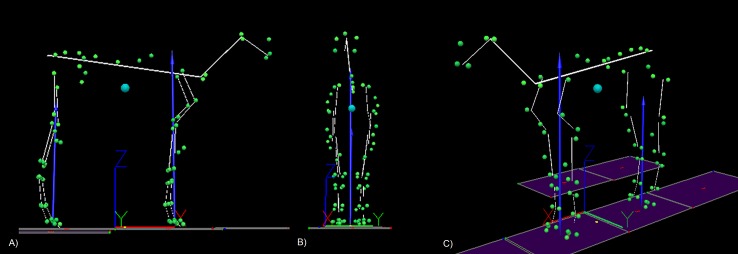
An example of the 25 segment model developed for each horse. (A) Sagittal plane view, (B) frontal plane view, (C) oblique view. The blue sphere represents the position of the centre of mass (COM). The cranio-caudal location of the COM is projected on to the ground and is shown as a yellow dot between fore and hindlimbs. The blue arrows represent the resultant ground reaction force vectors for fore and hind limbs. The green spheres represent the location of anatomical markers attached to the horse and the yellow lines represent the model segments. The origin and global coordinate system for the laboratory is depicted by the XYZ axes that can be seen underneath the horse model.

Kinematic and force data were recorded and prepared for analysis and a 25 segment model (see Fig. 1) was developed for each horse as described by Hobbs, Richards & Clayton (2014) and, in accordance with the results of that study, the segmental model COM was adjusted to the COP ratio during standing by shifting the trunk COM location. Consequently, the cranio-caudal segmental model COM location matched the body COP location during standing. The standing COP ratio (COP_STAND_) was determined as follows;

(1) \begin{eqnarray*}{\mathrm{COP}}_{\mathrm{STAND}}\mathrm{Ratio}= \frac{{\mathrm{GRFV }}_{F}}{{\mathrm{GRFV }}_{T}} \end{eqnarray*}

GRFV_*F*_ = forelimb vertical force; GRFV_*T*_ = summed forelimb and hindlimb vertical forces.

#### Temporal parameters

The timings of hoof contacts and lift offs were identified from the force plate data using a threshold of 50 N. One complete stride was used from each successful trial between successive right forelimb lift offs. Contralateral forelimb lift offs subdivided the stride into two diagonals; LFRH was the left forelimb and right hindlimb pair, and RFLH was the right forelimb and left hindlimb pair. Contacts of the diagonal pairs were classified as hind-first dissociation (also known as positive diagonal advanced placement), synchronous (also known as zero diagonal advanced placement) and fore-first dissociation (also known as negative diagonal advanced placement). Dissociation time for each diagonal pair was the time elapsing between fore and hind contacts, with the value of hind-first contacts being designated positive and fore-first contacts being designated negative.

#### GRF and moments parameters

GRFs were summed and COM-COP separation at time of zero fore-aft horizontal force (Tzero) identified for each step. Ground reaction force moments (MGRF) were calculated for each frame of data by summing the moments due to GRF from each limb component multiplied by their effective distance to the COM (Hobbs, Richards & Clayton, 2014), as defined in Eq. (2). A sign convention was established for moments, which is described when viewing the right side of the horse in the sagittal plane. A clockwise (nose-down) rotation about the COM was considered as positive and an anticlockwise (nose-up) rotation about the COM was considered negative. Tzero indicated the transition between absorbing and generating phases and mean moments were summed for each of these phases separately.

(2) \begin{eqnarray*}\mathrm{MGRF}={\mathrm{GRFV l}}_{lF}\hspace*{1em}\pm \hspace*{1em}{\mathrm{GRFLl}}_{vF}\hspace*{1em}\pm \hspace*{1em}{\mathrm{GRFV l}}_{lH}\hspace*{1em}\pm \hspace*{1em}{\mathrm{GRFLl}}_{vH}\end{eqnarray*}

*F* = forelimb, *H* = hindlimb

GRFV l_*l*_ = vertical GRF multiplied by fore-aft horizontal distance from the limb COP to the COM of the body

GRFLl_*v*_ = fore-aft horizontal GRF multiplied by vertical distance from the limb COP to the COM of the body.

#### Mass distribution parameters

COM and body COP locations along the cranio-caudal axis for each frame of data, and fraction of body weight on the forelimbs (Jz ratio), were calculated as reported by Hobbs, Richards & Clayton (2014). The COM location was determined using the segmental method, as described in Eq. (3)

(3) \begin{eqnarray*}\mathrm{COM}= \frac{\sum _{25}^{n}md}{M} \end{eqnarray*}

where COM = COM location relative to the origin of the laboratory coordinate system (LCS) (*m*) *m* = segment mass (kg)

*d* = distance of the segment COM relative to the origin of the LCS (*m*)

*M* =mass of the horse (kg).

The body COP location (*m*) was determined by taking moments relative to the origin of the LCS, as described in Eq. (4)

(4) \begin{eqnarray*}\mathrm{COP}= \frac{({\mathrm{GRFV l}}_{lF}+{\mathrm{GRFV l}}_{lH})}{({\mathrm{GRFV }}_{F}+{\mathrm{GRFV }}_{H})} .\end{eqnarray*}

The Jz ratio was calculated for each stance phase as described in Eq. (5)

(5) \begin{eqnarray*}\mathrm{Jz~ ratio}= \frac{{Jz}_{F}}{Jz} \end{eqnarray*}

where *Jz*_*F*_ = forelimb vertical impulse

*Jz* = summed forelimb and hindlimb vertical impulses.

The distance between the COM and COP was designated positive when the COM was ahead of (cranial to) the COP. Mean body COM and COP locations during each step were calculated using the ratio of the distance to the forelimb COP at Tzero divided by the distance between the forelimb and hindlimb COPs at Tzero (Hobbs & Clayton, 2013). This provided relative COM and body COP locations.

#### Postural parameters

**Figure 2:**
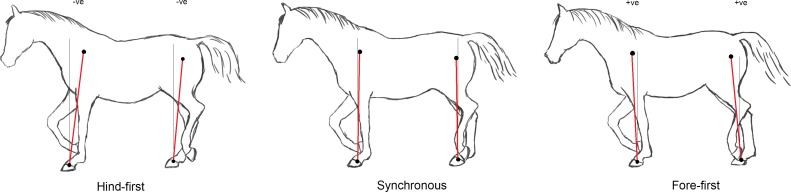
Illustration of mean limb postures for different dissociations during trotting. The sign of typical mean fore and hindlimb angles for hind-first and fore-first dissociations are shown with angles exaggerated to illustrate the different mean postures.

Limb angle was measured for each frame of data from the vertical to the line between the proximal and distal markers on the forelimb and hindlimb in the sagittal plane of the lab coordinate system. Limb retraction with the distal marker caudal to the proximal marker was designated positive. Limb protraction with the distal marker cranial to the proximal marker was designated negative (see Fig. 2). Trunk inclination was calculated as rotation about the trunk transverse axis in the lab coordinate system with nose-down from the horizontal as positive.

#### Speed parameters

The COM velocity was determined from the first derivatives of COM location. Relative (non-dimensional) COM velocity was calculated as,

(6) \begin{eqnarray*}{V}_{r}= \frac{V}{(lg)^{0.5}} \end{eqnarray*}

where *V* = velocity (ms^−1^); *l* = standing height (*m*); *g* = 9.81 (ms^−2^; acceleration of gravity).

#### Collisional parameters

Collisional angles were determined from the difference between the COM velocity angle and the orthogonally offset summed GRF angle in the sagittal plane as absolute values for each frame of data. Absorption angles (*ϕ*^−^) and generation angles (*ϕ*^+^) for each frame were negated, as collision angles are considered to be non-negative (Ruina, Bertram & Srinivasan, 2005). In dynamics a collision is defined simply as a discontinuity in the COM path (Bertram, 2013). Although collision losses are most easily visualized when considering passive interaction between colliding objects, active deflection of the COM due to actuation by the limbs during the generative phase of the stride does involve collision losses. In this case, however, the metabolic investment results in a net energy increase of the system. Net deflection (*ϕ*) over a step was calculated from (Ruina, Bertram & Srinivasan, 2005) by summing mean absorption and mean generation angles.

(7) \begin{eqnarray*}\phi ={\phi }^{-}+{\phi }^{+}.\end{eqnarray*}

## Data Analysis

For each step from forelimb lift off to the next forelimb lift off, mean values were calculated and tabulated for speed, Jz ratio, COM and COP location and trunk inclination. Mean values during absorption and generation were calculated for MGRF and collision angles. Net deflection was calculated as described in Eq. (7). Vertical GRF for the forelimbs and hindlimbs were integrated to obtain mean impulse. Metric values extracted at Tzero were the separation between the COM and body COP locations in the fore-aft direction and MGRF. Peak vertical GRF was determined and time to peak GRF was expressed as a % stance (individual limb) or % diagonal stance (limb pair). Trunk ROM was determined as the difference between minimum and maximum trunk inclination over the step. All calculations were performed in Visual 3D Professional v5.01.6 (C-Motion Inc.).

Metrics were imported into SPSS (IBM Corp.) for analysis. Data were tested for normality using a Kolmogorov–Smirnov test and were found to be normally distributed for speed-matched data. Simple bootstrapping was used on the speed range data, as the majority of parameters were not normally distributed. From the speed-matched dataset a 3 × 2 ANOVA was used to determine differences between dissociations (hind-first, synchronous, fore-first; combining data for the left and right diagonals) and diagonals (LFRH, RFLH; combining data for the three types of dissociations) for each variable separately with Bonferroni post hoc testing on significant differences in dissociation. Moderate-to-strong relationships (*R* > 0.55) between dissociation time and locomotion parameters, and relative COM velocity and locomotion parameters, were identified and compared between datasets. This was carried out using Pearson’s correlation for the speed-matched dataset and using Partial correlation, controlling for horse for the speed-range dataset. Significance was set at *P* < .05.

## Results

The frequencies of the different categories of diagonal dissociation in the speed-matched and speed-range data are shown in Table 1. For the speed-matched data, horses either contributed two hind first diagonals, two fore-first diagonals, two synchronous diagonals, one hind-first and one synchronous diagonal or one fore-first and one synchronous diagonal. The ensemble averages (mean ± s.d.) of each parameter from the speed-matched data in Table 2 are separated according to dissociation category (hind-first, synchronous, fore-first) and diagonal (LFRH, RFLH). Absolute variation in mean speed between diagonals for each trial was 0.03 ± 0.02 ms^−1^ indicating that these runs, though unconstrained, were as close to steady state as possible.

**Table 1:** Frequency of different dissociation categories. Number of LFRH-RFLH footfalls in each dissociation classification for speed-matched and speed-range datasets.

	HF-HF	HF-S	S-S	FF-S	FF-FF	FF-HF
Speed-matched						
Total	4	4	2	3	4	0
Speed-range						
Horse 1	6	2	0	1	0	1
Horse 2	10	0	0	0	0	0
Horse 3	1	2	2	2	3	0
Horse 4	1	0	1	1	7	0
Horse 5	0	1	0	5	2	2
**Total**	**18**	**5**	**3**	**9**	**12**	**3**

**Notes.**

HF-HF both hind-first HF-S one hind-first and one synchronous S-S both synchronous FF-S one fore-first and one synchronous FF-FF both fore-first HF-FF one hind-first and one fore-first

**Table 2:** Locomotion parameters grouped by dissociation category and diagonal (speedmatched, *n* = 17 horses). No significant differences were found for diagonal. No interactions between dissociation and diagonal were found. Significance (Sig.) for each parameter between dissociation classifications is given, with significant differences (*P* < .05) highlighted in bold. Superscript letters denote Post hoc comparisons between dissociation pairs, where a = significantly different (*P* < .05) to hind-first; b = significantly different (*P* < .05) to synchronous; and c = significantly different (*P* < .05) to fore-first.

	LFRH			RFLH			
	Hind-first	Synchron-ous	Fore-first	Hind-first	Synchron-ous	Fore-first	Sig.
*n*	5	6	6	7	5	5	
**Speed parameters**							
COM Velocity (ms^−1^)	3.30 (0.08)	3.22 (0.18)	3.26 (0.30)	3.27 (0.13)	3.28 (0.17)	3.21 (0.17)	.749
Relative COM Velocity	0.864 (0.04)	0.837 (0.04)	0.852 (0.07)	0.854 (0.04)	0.857 (0.06)	0.833 (0.05)	.713
**Mass Distribution Parameters**							
Mean COM location	0.419 (0.02)	0.433 (0.03)	0.418 (0.02)	0.429 (0.03)	0.419 (0.02)	0.422 (0.02)	.854
Mean COP location	0.416 (0.03)^bc^	0.350 (0.02)^ac^	0.315 (0.03)^ab^	0.402 (0.04)^bc^	0.360 (0.02)^ac^	0.318 (0.01)^ab^	<**.001**
Jz ratio	0.571 (0.02)	0.571 (0.01)	0.589 (0.02)	0.578 (0.01)	0.570 (0.01)	0.581 (0.01)	.071
COM-COP separation at Tzero (m)	0.03 (0.03)^c^	0.03 (0.01)	0.05 (0.02)^a^	0.02 (0.02)^c^	0.05 (0.02)	0.05 (0.02)^a^	**.007**
**GRF Parameters**							
Peak GRFV F (N/kg)	11.14 (0.82)	11.34 (0.60)	10.78 (0.40)	11.20 (0.77)	11.22 (0.57)	10.84 (0.53)	.212
Peak GRFV H (N/kg)	8.32 (0.39)^b^	9.39 (0.84)^a^	8.89 (0.84)	8.42 (0.67)^b^	9.39 (0.61)^a^	9.11 (0.38)	**.004**
Impulse F (Ns/kg)	1.84 (0.12)	1.97 (0.10)	1.96 (0.13)	1.88 (0.13)	1.94 (0.13)	1.92 (0.06)	.162
Impulse H (Ns/kg)	1.38 (0.11)	1.48 (0.08)	1.37 (0.17)	1.37 (0.06)	1.47 (0.14)	1.38 (0.07)	.078
**Moments parameters**
MGRF at Tzero (Nm/kg)	0.57 (0.51)^bc^	0.75 (0.20)^a^	0.94 (0.26)^a^	0.43 (0.43)^bc^	1.19 (0.43)^a^	1.14 (0.35)^a^	**.004**
MGRF absorption (Nm/kg)	0.39 (0.33)^c^	0.17 (0.23)	–0.07 (0.18)^a^	0.30 (0.19)^c^	0.28 (0.30)	0.09 (0.15)^a^	**.007**
MGRF generation (Nm/kg)	0.08 (0.19)	0.12 (0.15)	0.19 (0.25)	–0.05 (0.26)	0.33 (0.31)	0.27 (0.25)	.078
**Collisional Parameters**
Net deflection (rad)	0.48 (0.04)^b^	0.53 (0.03)^a^	0.51 (0.04)	0.49 (0.04)^b^	0.53 (0.03)^a^	0.51 (0.02)	**.017**
Mean absorbing angle (rad)	0.25 (0.02)^b^	0.27 (0.02)^a^	0.26 (0.03)	0.25 (0.03)^b^	0.27 (0.02)^a^	0.26 (0.01)	**.044**
Mean generating angle (rad)	0.23 (0.02)	0.25 (0.01)	0.25 (0.02)	0.24 (0.01)	0.26 (0.02)	0.25 (0.02)	.070
**Postural Parameters**
Mean limb angle F (deg)	–1.27 (1.33)^c^	0.58 (0.96)	2.16 (2.66)^a^	–0.09 (1.04)^c^	–0.15 (2.59)	2.29 (2.23)^a^	**.004**
Mean limb angle H (deg)	–1.77(1.08)^c^	–1.41 (1.45)^c^	1.10 (2.46)^ab^	–2.62 (0.96)^c^	–0.64 (1.50)^c^	0.20 (1.16)^ab^	**.001**
Trunk ROM (deg)	0.93 (0.43)	0.95 (0.48)	1.40 (0.56)	1.27 (0.43)	0.92 (0.51)	1.11 (0.46)	.308
Mean trunk inclination (deg)	8.72 (1.64)^c^	10.05 (1.26)	10.36 (0.85)^a^	9.12 (1.86)^c^	9.89 (0.95)	10.45 (0.71)^a^	**.034**
**Temporal Parameters**							
Time to peak GRFV F (% diagonal stance)	47.82 (3.24)^b^	43.14 (2.05)^a^	47.02 (3.01)	48.38 (3.37)^b^	45.33 (2.10)^a^	46.58 (4.19)	**.009**
Time to peak GRFV H (%diagonal stance)	42.31 (3.20)	40.39 (1.70)	45.47 (3.52)	43.31 (2.96)	42.35 (0.69)	42.88 (2.12)	.057
Time to peak GRFV F (% stance)	45.66 (2.24)	43.14 (2.05)	47.02 (3.01)	45.39 (2.23)	45.33 (2.10)	46.58 (4.19)	.103
Time to peak GRFV H (% stance)	44.73 (3.01)	44.13 (0.86)	46.30 (3.28)	45.97 (2.33)	45.37 (2.29)	44.28 (1.53)	.790

**Figure 3:**
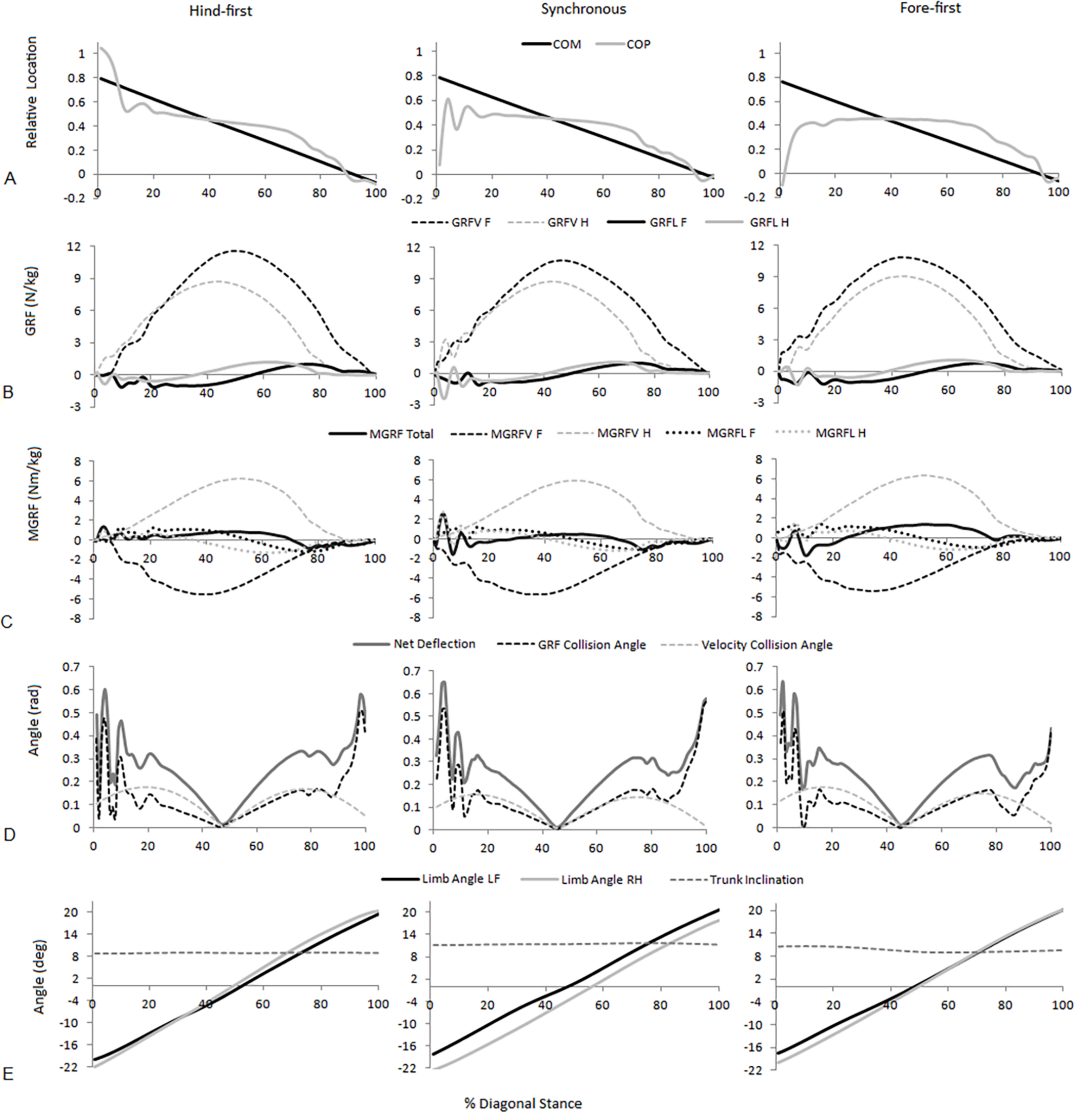
Examples of significant parameters from typical hind-first, synchronous and fore-first dissociation patterns when speed-matched. (A) Relative COM and COP location from the forelimb COP position at Tzero; (B) Vertical (GRFV) and longitudinal (GRFL) GRFs for the fore and hindlimbs (N/kg); (C) total and fore and hindlimb components of ground reaction force moments (MGRF) (Nm/kg); (D) GRF collision angle, velocity collision angle and net deflection (rad); (E) Fore and hindlimb angles and trunk inclination (degrees).

For speed-matched data the ANOVA found significant (*P* < .05) differences between dissociation categories for four functional parameters (peak vertical hindlimb GRF, net deflection, mean absorbing angle, time to peak vertical forelimb GRF (% diagonal stance)), three postural parameters (mean forelimb angle, mean hindlimb angle, mean trunk inclination) and four balance parameters (mean COP location, COM-COP separation at Tzero, MGRF at Tzero, MGRF during absorption). Of those, significant differences between all three dissociation categories were found for only one parameter, mean COP location (hind-first vs synchronous; *P* < .001, hind-first vs fore-first; *P* < .001, synchronous vs. fore-first; *P* = .006). Figure 3 shows temporal data of significant parameters for one step for one horse producing hind-first, one horse producing synchronous and one horse producing fore-first dissociation. No significant differences (*P* > .05) were found for diagonal (LFRH versus RFLH) and there were no significant interactions (*P* > .05) between type of dissociation and diagonal. In the speed-matched data horses with hind-first dissociation tended to have more protracted mean limb angles together with a more rearward location of the COP and vice versa for fore-first dissociation (Table 2).

**Figure 4:**
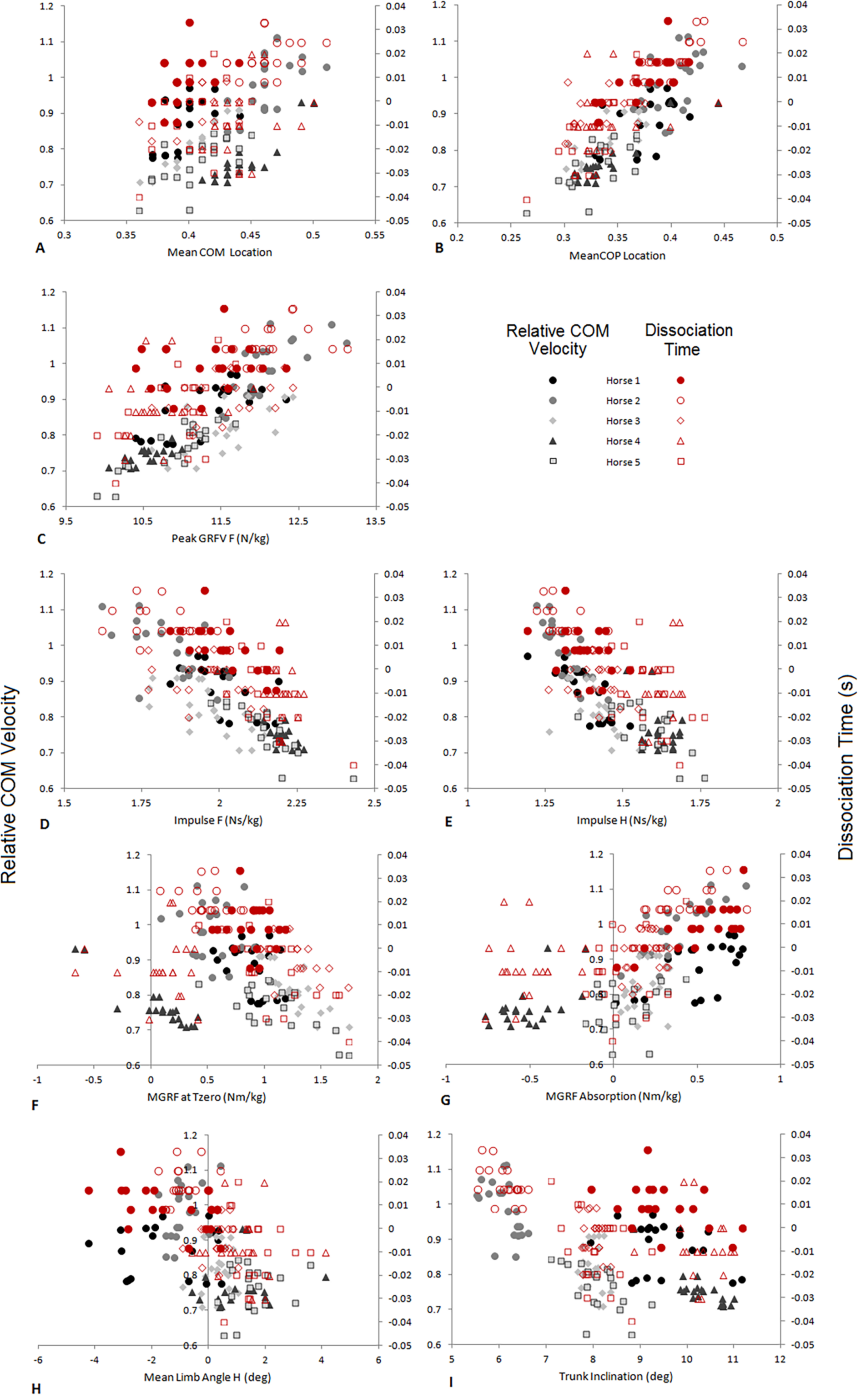
Comparison of relative COM velocity (black/grey-left vertical axis) and Dissociation Time (s) (red-right vertical axis) to variables identified in Table 2 for speed range data (*n* = 5 horses × 10 trials × 2 steps). (A) Mean COM Location, (B) Mean COP Location, (C) Peak GRFV F (N/kg), (D) Impulse F (Ns/kg), (E) Impulse H (Ns/kg), (F) MGRF at Tzero (Nm/kg), (G) MGRF Absorption (Nm/kg), (H) Mean Limb Angle H (deg), (I) Trunk Inclination (deg). The data from each horse is identified with the same symbol, so for each horse a different symbol is used.

**Figure 5:**
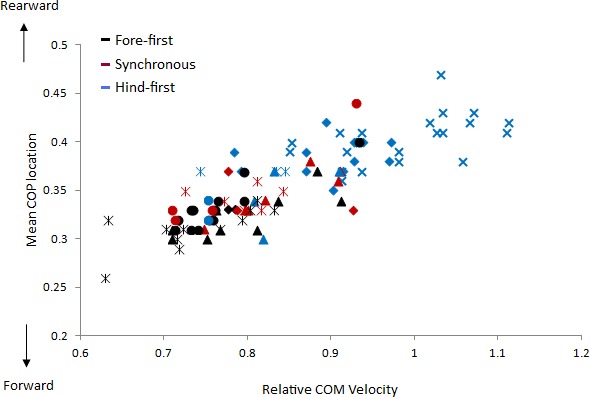
Comparison of relative COM velocity to COP location for speed range data (*n* = 5 horses × 10 trials × 2 steps). Each step is classified with respect to its dissociation using colours where black = fore-first, red = synchronous and blue = hind-first. The data from each horse is identified with the same symbol, so for each horse a different symbol is used.

The velocity of the speed-range dataset was between 2.43 and 4.23 ms^−1^. Relationships between locomotion parameters, dissociation time and relative COM velocity that were moderate to strong (*R* > .55) for either dataset are shown in Table 3 and Fig. 4. The relationship between dissociation time and relative COM velocity for speed-matched data was *R* = .119 and for the speed range data was *R* = .774. Mean COP location had the strongest relationship with dissociation time when data were speed-matched, but also had a strong relationship with relative COM velocity over the speed range (Table 3). MGRF at Tzero, MGRF during absorption and mean hind limb angle shared only moderate relationships with the speed-matched dataset, whereas trunk inclination had moderate relationships in both datasets. Conversely, forelimb vertical GRF had a strong relationship with dissociation time over the speed range (*R* = .594; *P* < .01) but not when speed-matched (*R* = .145; *P* > .05). Although only small differences in speed were recorded between horses in the speed-matched dataset, fore and hindlimb impulses were still found to have moderate to strong relationships with relative COM velocity, (with forelimb impulses just outside of the threshold criteria). None of the other functional, postural or balance parameters had moderate to strong relationships in either dataset. Figure 5 depicts mean COP location against relative COM velocity for the speed-range data showing the dissociations used by each horse for each step.

**Table 3:** Comparison of moderate to strong relationships of locomotor parameters to dissociation time and speed for speed matched and speed-range data. Correlation coefficients for speed-matched data (Pearson correlations, *n* = 17 horses × 1 stride per horse) compared to speed range data (Partial correlation controlling for horse, *n* = 5 horses × 10 strides per horse) where a moderate to strong (*R* < .55) relationship was found in either dataset.

	Speed-matched		Speed Range	
Parameter	Dissociation time	Relative velocity	Dissociation time	Relative velocity
Mean COM location	–0.103	–0.364^*^	–0.584^**^	–0.710^**^
Mean COP location	–0.920^**^	–0.167	–0.857^**^	–0.764^**^
Peak GRFV F	0.145	0.052	0.594^**^	0.789^**^
Impulse F	–0.384^*^	–0.522^**^	–0.652^**^	–0.745^**^
Impulse H	0.011	–0.570^**^	–0.512^**^	–0.695^**^
MGRF at Tzero	–0.565^**^	–0.342^*^	–0.330^*^	–0.298^*^
MGRF absorption	0.567^**^	–0.155	0.429^**^	0.420^**^
Mean limb angle H	–0.611^**^	–0.294	–0.262^*^	0.030^*^
Mean trunk inclination	–0.571^**^	–0.107	–0.566^**^	–0.654^**^

**Notes.**

* Significant correlation *P* < .05.

** Significant correlation *P* < .01.

## Discussion

This study investigated the effects of diagonal dissociation on locomotion parameters related to function, posture and balance in horses trotting at the same speed and across a modest range of naturally occurring trotting speeds. We hypothesised that: (1) hind-first dissociation would increase with speed; (2) diagonal dissociation would reduce collisional energy losses; and (3) within horse dissociation predominance would be evident at preferred trotting speed. Mean COP location varied with dissociation in horses trotting at the same speed. The COP location also changed with increasing speed, accompanied by an increase in peak forelimb vertical force. Hypothesis (1) was partially supported, as hind-first dissociation increased with speed, but between horse variations in dissociation pattern could be contributing to this finding. Hypothesis (2) was partially supported since net collisional losses were reduced during absorption when hind-first was compared to synchronous dissociation at the same trotting speed. There was some evidence to support within horse dissociation predominance (hypothesis 3), but further work is needed to clarify this as a strong relationship is evident between speed and dissociation time.

For a cursorial mammal with relatively long limbs, a high COM position, and limbs that move primarily in a parasagittal plane, balancing pitching moments will be an important stability consideration. There are three fundamental motor control strategies available to accomplish this. These are adjustments of (i) relative fore-aft contact timing, i.e., diagonal dissociation (as shown by Weishaupt et al. (2009)); (ii) foot contact position (Lee, Bertram & Todhunter, 1999); and (iii) fore-aft vertical force distribution (Lee, Bertram & Todhunter, 1999). The first successful trotting quadrupedal robot utilized a control strategy with synchronized diagonal contacts with equal fore-aft contact forces and adjusted contact position to maintain fore-aft stability (Raibert, 1986; Raibert, 1990). In trotting dogs, Lee, Bertram & Todhunter (1999) found that moderate fore-aft moments were balanced primarily by adjusting fore-aft contact forces with relatively consistent fore-aft contact position. Lee, Bertram & Todhunter (1999) also found that subtle differences in contact timing could be detected between breeds (Labrador retriever vs. greyhound) and attributed this largely to differences in body form and mass distribution (a conclusion consistent with experiments with quadrupedal robots, Raibert, 1990). The current study provides evidence that horses subtly employ all three of these strategies when trotting at constant speed and that this is likely linked to balance maintaining parameters.

The first strategy, diagonal dissociation, altered the mean COP location in horses trotting at the same speed (Table 2). The subtle difference in timing at the beginning of the stance phase changed the inter-limb timing of force production and consequently the relative fore-hind force contributions throughout stance. The mean COM location, which can also influence force distribution patterns, was not different with dissociation. Alexander (2002) suggested that dynamic stability in quadrupeds may be achieved by altering the timing of peak force production within limbs, thereby changing the effective value of the distance from the COM to that limb. The temporal parameters (Table 2) show that the timing of peak force production is only altered in hind-first dissociation in which peak forelimb force occurs later during diagonal stance. This affects the COP location by causing a more gradual change in ratio towards the forelimb (Fig. 3). Dissociation has also been shown to change with lameness. When inducing a fore hoof lameness, fore-first dissociation time increased progressively with the degree of lameness on both diagonals (Buchner et al., 1995). In mild to moderate forelimb-induced lameness, increasing forelimb stance duration was found to be the main mechanism that the horse uses to reduce GRFV whilst maintaining impulse (Weishaupt et al., 2006). The effect of dissociation time on balance in lame horses is yet to be fully explored.

The second strategy was also evident at constant speed, as forelimb and hindlimb mean angles varied between dissociations. For hind-first dissociation there was a tendency for both limbs to be more protracted which allows the hindlimb to step further under the body at contact and the forelimb to leave the ground in a more vertically oriented position at lift off. Horses with fore-first dissociation appeared to adopt a more ‘falling forwards’ posture, as COM-COP separation at Tzero was more positive for fore-first dissociation and mean limb angles were more retracted compared to hind-first dissociation (Table 2). With this posture the COM and gravity may be used to develop greater forward and downward moments prior to Tzero to balance the earlier negative MGRF moments during braking. This difference is illustrated in Fig. 3, where the positive moments prior to Tzero were greater with fore-first dissociation. This strategy could be likened to the theory of Pose^®^ running in humans, where landing with a vertically aligned COM and COP allows gravitational moments to be used as the main force that moves the COM forwards (Romanov & Fletcher, 2007). Running economy was, however, not improved using this technique compared to heel-toe running (Fletcher, Romanov & Bartlett, 2008). Further work might consider whether oxygen consumption is optimized with predominant dissociation patterns in trotting.

The first strategy, dissociation, also shifted as speed moderately increased. Although an increase in speed need not involve a change in moment, provided speed is constant stride to stride, strong correlations between dissociation time, speed and COP location were evident. The uneven fore-aft mass distribution of the horse (with the majority of the mass carried by the fore quarters), likely results in residual moments over the stride cycle that will be greater at faster trotting speeds. One interpretation of the current result is that dissociation contributes to the mitigation of these moments as speed increases, but the pattern of shift (from fore first to synchronous or synchronous to hind first) will be dependent on the specific body proportions of the individual (and also influenced by the particular subject’s reliance on aspects of the alternative strategies).

Peak force increased with speed in the forelimb (Fig. 4), but not in the hindlimb, which was also reported by Dutto et al. (2004) at moderate speeds. If dissociation was not used to increase nose-down moments during absorption, then nose-up residual moments could accumulate and challenge balance under these conditions. Other strategies to manage pitching moments are reported when speed is increased beyond energy efficient thresholds (Hoyt & Taylor, 1991). These include racing trotters moving at high speed which show a stronger relationship between speed and peak vertical force in the hindlimbs compared to the forelimbs (Crevier-Denoix et al., 2014). However, these horses were pulling a sulky which is likely to affect force generation and distribution between limbs. Deuel & Park (1990) also reported a range of dissociations from hind-first to fore-first in horses performing extended trot, so individual predominance is still evident at higher speeds in highly trained horses. In order to produce a larger hindlimb force one might expect the hindlimb would either be closer to the COM during peak force production (in order to add vertical impulse to support body weight while limiting the contribution to pitching moment) or that there would be an increase in limb stiffness thereby producing a larger reaction at the ground. Increased hindlimb muscle activity at 6 ms^−1^ compared to slower trotting speeds has been reported (Robert et al., 2002), which was attributed to applying greater force during hip extension. This suggests that the third balancing strategy will be evident at higher speeds.

It has been argued that collision-like losses associated with the limbs deflecting the COM are important in determining, and consequently in interpreting, gait dynamics (Bertram, 2013; Bertram & Gutmann, 2009; Ruina, Bertram & Srinivasan, 2005). Despite the small magnitude of the temporal dissociations of the footfalls, net collisional losses and collisional losses during absorption were significantly greater with synchronous compared to hind-first dissociations at the same speed. Hind-first dissociation also produced positive MGRF during absorption. These findings illustrate the profound effects of limb sequencing, where individual limbs can be thought of much like the spokes of a rolling rimless wheel which helps to distribute the angular deflection changes, thereby reducing collisional losses (Ruina, Bertram & Srinivasan, 2005). The advantages of limb sequencing in reducing collisional losses have mainly been reported for gaits that have a sequential footfall pattern, cantering in ring tailed lemurs (O’Neill & Schmitt, 2012), galloping in horses and cheetahs (Bertram & Gutmann, 2009), and walking and galloping, but not trotting, in dogs and goats (Lee et al., 2011). Collisional losses appear high during trotting (Lee et al., 2011), but total mechanical cost would likely be even higher to move at the same speed with a different gait (if the equine trot functionally resembles the human run; Srinivasan & Ruina, 2006). The trot is usually considered to have synchronous diagonal contacts but our findings indicate that there is scope for collisional losses in trot to be mitigated to some degree by diagonal dissociation.

Stabilizing the trunk also appears to be important in horses during trotting, as a very small trunk ROM was found in this study, which concurs with Dunbar et al. (2008) and Buchner, Obermüller & Scheidl (2000). In the quest for spinal stability during trotting, the epaxial and hypaxial muscles are activated to reduce vertical thoracic and lumbar spinal excursions (Robert et al., 2002), while splenius and semispinalis capitis provide postural stability of the cervical spine (Gellman, Bertram & Hermanson, 2002). Diagonal dissociations may then be used to manage COP excursions, which minimize pitching moments to provide rotational stability. In human walking it was suggested that trunk angular momentum is highly regulated by the central nervous system (Popovic, Hofmann & Herr, 2004). Based on our findings, quadrupedal trotting may have similar requirements. Trunk inclination was influenced by speed; when speed increased the mean trunk angle decreased slightly to a more nose-up posture. However, a relationship between trunk inclination and dissociation time was also evident in the speed-matched dataset, so hind-first dissociations were also associated with a more nose-up trunk posture. Weishaupt et al. (2009) found an association between a more elevated head and neck position and increased hind-first dissociation time when comparing passage to collected trot. In this case passage was performed at a slower speed than collected trot, so an inverse relationship was evident compared to the speed-range dataset. It is also interesting to note that trunk inclination in Greyhounds compared to Labrador Retrievers is likely to vary with dissociation pattern in a similar manner (Bertram et al., 2000). Further work is needed to investigate motor control strategies used in horses performing higher level movements.

One of the study aims was to evaluate potential reasons for adopting a predominant diagonal dissociation pattern within an individual horse. The choice of preferred speed may influence the habitual dissociation pattern used by the individual and this may also relate to maximizing energy efficiency at that speed whilst maintaining pitch stability. Inter-breed differences in peak vertical forelimb GRF between Warmbloods and Quarter Horses have been attributed to conformation and gait differences (Back et al., 2007). It is likely that diagonal dissociation will also vary in those breeds, although from this study it appears that dynamic rather than static posture is a more important determinant. Given the multiple strategies available to the animal and the subtle relationship between them identified in this study, it is evident that more work is needed in horses and other species to confirm this observation. In addition, this study only covered a moderate range of speeds occurring within the natural (energy efficient) range for trotting horses (Hoyt & Taylor, 1991). Different force production patterns have been reported at higher speeds in harness horses (Crevier-Denoix et al., 2014) suggesting that further work is needed to investigate the mechanical effects at speeds beyond those performed by the general equine population.

## Conclusions

Dissociation patterns vary between horses trotting at the same speed, but speed and dissociation time are also intrinsically linked. When comparing data within and across a range of speeds subtle differences in dissociation could be explored to investigate why individual horses use different dissociation patterns. The evidence presented suggests that at moderate speeds horses use dissociation to maintain trunk pitch stability by managing the COP location. This is likely due to body proportion differences but could also be influenced by the motor control strategy utilized by the individual animal. Both hind-first and fore-first dissociations may have mechanical advantages over synchronous contacts in certain circumstances. As trotting speed increases, forelimb vertical peak force increases and dissociations tend towards hind-first, principally to shift the COP caudally and control trunk pitching moments.

## Abbreviations

COM: Centre of mass
COP: Centre of pressure
g: Acceleration due to gravity
GRF: Ground reaction force
GRFV: Vertical ground reaction force
Jz ratio: Fraction of body weight on the forelimbs
l: Standing height/Moment arm
LF: Left forelimb
LH: Left hindlimb
MGRF: Ground reaction force moments
RF: Right forelimb
RH: Right hindlimb
ROM: Range of motion
Tzero: Time of zero longitudinal force
V: Velocity
Vr: Relative COM velocity

## Glossary

Dissociation pattern: The sequence of footfalls of a diagonal limb pair used by an individual horse during trotting gaits.
Dissociation time: The length of time between forelimb and hindlimb ground contact.
Fore-first dissociation: A diagonal footfall sequence where the forelimb contacts the ground before the hindlimb. Otherwise known as negative diagonal advanced placement.
Hind-first dissociation: A diagonal footfall sequence where the hindlimb contacts the ground before the forelimb. Otherwise known as positive diagonal advanced placement.
Nose up pitch rotation of the trunk: Pitching rotation of the body that would lift the forehand.
Synchronous footfalls: A diagonal footfall sequence where the forelimb and hindlimb contacts the ground at the same time.

## Additional Information and Declarations

### Data Availability

The following information was supplied regarding data availability: The raw motion files (in CMO format) and video animations of the three dissociation classifications are available at: 10.17030/uclan.data.00000040. CMO files can be viewed using a free CMO Reader available to download from C-Motion Inc. at: http://www2.c-motion.com/free. Excel spreadsheets for both datasets of the metrics can be found at: 10.17030/uclan.data.00000040.
